# Access to pediatric extracorporeal membrane oxygenation: a geospatial analysis of the racial/ethnic composition of areas with and without access

**DOI:** 10.1186/s12939-025-02571-7

**Published:** 2025-07-01

**Authors:** Zoel A. Quiñónez, Kathleen Ryan, Tristan D. Margetson, Elisabeth Grosvenor, Charlotte D. Smith, Laura M. Diaz, Angel Benitez-Melo, Seth Hollander, Danton Char

**Affiliations:** 1 Department of Anesthesiology, Perioperative and Pain Medicine, Stanford University School of Medicine, 300 Pasteur Dr Rm H3580 MC, Stanford, CA 5640, 94305 USA; 2 Graduate Division, School of Public Health, University of California, Berkeley, Berkeley, CA USA; 3 Lucile Packard Children’s Hospital, Department of Pediatrics, Division of Cardiology, Stanford University Medical Center, Palo Alto, CA USA; 4 Betty Irene Moore Children’s Heart Center, Lucile Packard Children’s Hospital, Stanford University Medical Center, Palo Alto, CA USA; 5 Division of Environmental Health Sciences, School of Public Health, University of California Berkeley, Berkeley, CA USA; 6 Department of Biology, University of San Diego, San Diego, CA USA

**Keywords:** Disparity, Equity, Extracorporeal membrane oxygenation, Geospatial, Healthcare access

## Abstract

**Background:**

We propose that all communities should have access to lifesaving technologies like pediatric extracorporeal membrane oxygenation (ECMO), and that distance is one actionable component to accessibility. We chose to examine whether geographic access by distance to pediatric ECMO differs by race/ethnicity for populations historically excluded from health services and technologies.

**Methods:**

Population data was obtained from the US Census Bureau’s American Community Survey. Pediatric ECMO program data was obtained from the Extracorporeal Life Support Organization Registry. We compared the proportion of individuals that are American Indian/Alaska Native, Black/African American, Hispanic/Latina(o), or White that live within and outside of a 200-mile distance from pediatric ECMO programs.

**Results:**

43% of the total US land area falls outside of the US catchment area for pediatric ECMO; and 4.91% of the US population (or 16,433,563 persons) does not have access to a Pediatric ECMO center. One of every four individuals that identify as American Indian/Native American, one of every 100 who identify as Black/African American, one of every 12 that identify as Hispanic/Latina(o), and one of every 21 that identify as White live outside of the pediatric ECMO catchment area for the United States.

**Conclusions:**

American Indian/Native Americans and Hispanic/Latina(o)s lack access to pediatric ECMO by proximity. While Black/African Americans live close to ECMO programs, previous studies show that this population has less access to primary and specialized care. Distance is one actionable measurement that should be used to extend access to medical technologies for populations that have historically been excluded.

## Background

Extracorporeal Membrane Oxygenation (ECMO) is a mechanical circulatory rescue for patients in respiratory and/or circulatory failure that oxygenates a patient’s blood when the lungs have failed or circulates blood through the body when a patient’s heart has failed [1]. For many causes of respiratory or cardiac failure, ECMO has become a life-saving standard of care [1–3]. An ECMO program requires a highly specialized team including surgeons, cardiologists, intensivists, anesthesiologists, nurses, and perfusionists, as well as protocols for implementation, transport to the ECMO center, and an infrastructure for ECMO use [4–6]. Consequently, like many advanced medical technologies, ECMO has been largely limited to tertiary and quaternary care centers. ECMO is now integrated into the American Heart Association’s Pediatric Advanced Life Support recommendations and is increasingly becoming a standard of care intervention for resuscitation efforts more broadly [1–3, 7]. Pediatric ECMO data demonstrate that ECMO is usually initiated under time pressure in response to deteriorating clinical conditions, and that timing of initiation is important for successful outcomes [1]. While many factors contribute to survival, proximity to ECMO expertise is clearly important. Given its increasing integration as a standard of care life-saving technology, we should examine gaps in access to pediatric ECMO, particularly regarding populations historically excluded from specialty care, so that we can address inequities in access.

Previous geospatial analysis demonstrated that rural populations are at a greater distance to centers with pediatric ECMO capabilities [8–10]. Others have demonstrated that pediatric patients on ECMO support can be safely transported from remote locations, or from low volume center, to referral institutions [11, 12]. But we do not yet know what patients that require ECMO urgently or emergently have access to it based on where they live; nor do we have an complete understanding of patient populations that have access to it and those that do not. In looking to provide life-saving technologies like pediatric ECMO, we should identify regions that lack access, as well as identifying communities that have been excluded from access to initiate specific targeted programs to increase service to these areas and communities. For instance, American Indians have been disproportionately burdened by infectious disease transmission and death from infections like hantavirus and COVID-19, at least in part due to the lack of infrastructure needed to respond to disease outbreaks and lack access to primary, non-emergent, obstetric and specialty care [13–20]. 

Here we focus on how racialized geographies define access for populations in the U.S. whose ancestry is rooted those communities that predated the arrival of Europeans (American Indian, including Latina(o)s) or who were forcefully taken from their land and brought to the U.S. (African Americans) as part of the colonial project. While other racialized and marginalized groups suffer impacts of exclusion (like Asian, Pacific Islanders, Irish, German, Italian, LGBTQ+), analysis of these populations, while important, falls outside of the scope of this analysis.

Understanding this geographic history of racialized groups as a factor in their lack of access to technologies is imperative given that the establishment of racialized geography was a deliberate process that might otherwise seem like a circumstantial reality. American Indians, for instance, were forcefully relocated away from the expanding European-American society through the Indian Removal Act of 1830 [21]. As such, American Indians remain the minority group with the lowest proportion living in urban areas despite later attempts to compel them to urbanize and assimilate through “Indian termination.” [22, 23].

For Latina(o)s, whose ancestry is comprised of American Indian and the African diaspora, as well as that of the European, more recent US interventionism in Latin America has also defined these racialized geographies [24, 25]. Extractive economic policies throughout Latin America by the U.S., the U.S. support of repressive governments in Latin America, and their involvement in regime change or attempted regime change have created instability as an additional driving force for migration from Latin American countries [24–26]. This can be seen in the 59-fold increase in Venezuelans detained at the border, 42-fold increase Nicaraguans detained, and 13-fold increase in Cubans detained between 2020 and 2023 an examples of this [26]. Labor policies, like the Bracero program, also created a pipeline for agricultural work, and created a pattern of labor-based migration, some of it to rural areas, for populations coming from Latin America [18, 27]. And while Latina(o)s have historically been the most urbanized racialized minority group, documented and undocumented people from Latin America continue to be shuttled into cheap labor roles within agriculture and other industries [27, 28]. 

While African Americans largely resided in rural areas before the 20th century, this changed with their migration out of the South and rural communities and towards urban centers during the late 19th and 20th centuries to escape southern segregationist laws and Jim Crow policies, as well as to employment shifts towards industrialization and militarization [29]. Thus, while geography serves as one structure by which communities have or lack access, other factors, such as insurance status, income, the cost of care, English proficiency, and provider bias can impact access to costly medical technologies [30]. 

Also contributing to these disparities is the impact of direct private and public under-resourcing, “cost-effectiveness” strategies, the setting of standards by regulatory bodies that favor well-resource White-serving institutions, and purposeful exclusion of Black, American Indian and Latina(o) communities of adequate medical, education and other social and public services [16, 17, 24, 30–39]. Nevertheless, geography, and thus proximity to a center that provides this lifesaving care, is one actionable component of this access.

We evaluate access to pediatric ECMO for American Indian/Alaska Native, Black/African American, Hispanic/Latina(o) and White populations by distance. We first defined a total catchment area for the United States, as well as the proportion of persons defined as that have access based on distance to a center. We hypothesized that populations in areas within a 200-mile driving distance to an ECMO center – the estimated catchment area for our and other children’s hospitals’ ECMO programs – differ in race/ethnicity from those without access [40].

## Methods

This was a population-level cross-sectional study of publicly available US Census Bureau data, as well as from a public registry of programs providing extracorporeal membrane oxygenation for children [41]. The study does not meet the definition of human subject research, as all data is publicly available and not identifiable, thus there was no need for IRB approval per Stanford University’s Research Compliance Office. Addresses of programs with pediatric ECMO capability were obtained from the Extracorporeal Life Support Organization Registry (ELSO) and geocoded using the World Geocoding Service in ArcGIS Pro v 3.2.2 (ESRI, Redlands, CA) [42]. All geospatial and statistical analysis were performed using ArcGIS Pro v 3.2.2 (ESRI, Redland, CA).

Our primary question is whether access exists for patients who are not yet supported by ECMO, but who either have emergent need for it or will require it because of a worsening condition. Our assessment focuses on ground transportation given that the distances that fixed- or rotary-wing aircraft can travel are not consistent, and that weather causes significant variability in its use across times/seasons and across different regions of the country. At our institution, 96.5% of ECMO transports are ground, with 2.5% rotary-wing and 1% fixed-wing. In this regard, we focus on two primary factors to define access to ECMO. First is the distance that programs determine that they can safely travel to pick up a patient that is not yet on ECMO for transport back to the pediatric ECMO center, or for the specialized team to travel to a hospital to place a patient on ECMO and transport them back with the team. The second factor is the distance a parent might be willing to transport their child when there is an urgent medical need that may come to require ECMO.

For that reason, we chose catchment area rather than spatial accessibility given that this is a better reflection than measures average distance to a set of providers, or models of provider influence or supply-demand analyses like two-step floating catchment area. We also assessed the average distance to the nearest pediatric ECMO center by race/ethnicity.

To define the catchment area for ECMO, we applied a 200-mile driving distance from each ECMO program. This distance is the catchment area for Lucile Packard Children’s Hospital (Stanford University), reflects the self-reported catchment area of other programs, and was determined from discussion with providers at other centers [40]. While 200 miles is somewhat arbitrary dividing line when considering programs that might be slightly closer or farther, this accessibility was based on expert opinion and to the exclusion of transport by aircraft that does not substantially contribute to ECMO transport in children [12, 43, 44]. 

To obtain census tract level data for individual race/ethnicity categories as defined by the American Community Survey we used the “ACS Race and Hispanic Origin Variables – Boundaries” available from esri_demographics to identify counts for individuals that identify as American Indian/Alaska Native alone, Black/African American alone, Hispanic/Latina(o) alone, or White alone [45]. 

For the catchment area analysis, we used ArcGIS Pro to generate service areas using the defined 200-mile driving distance for each program. We dissolved these service areas and performed a union between this layer and the “ACS Race and Hispanic Origin Variables – Boundaries” layer. For those census tracts that fall on the dividing line between having access and not having access, we performed areal interpolation to apportion the counts of individual race and ethnicity categories to each side of the line. To assess differences, we calculated proportions by combining all census level counts for areas within and outside of the defined access area. Z-scores for differences in proportions were calculated within ArcGIS Pro and R [R Core Team, Vienna] used to determine *p*-values [46]. 

For the distance to the nearest pediatric ECMO center, we generated centroids for each US census tract and assessed the geodesic distance from that centroid to the nearest ECMO center in US Survey miles using the “Near” analysis tool in ArcGIS Pro. We then weighted these distances by population numbers for each census by race/ethnicity. We then summed these census tract-level distances by race/ethnicity to calculate the average distance to the nearest pediatric ECMO center by race/ethnicity.

## Results

**Fig. 1:**
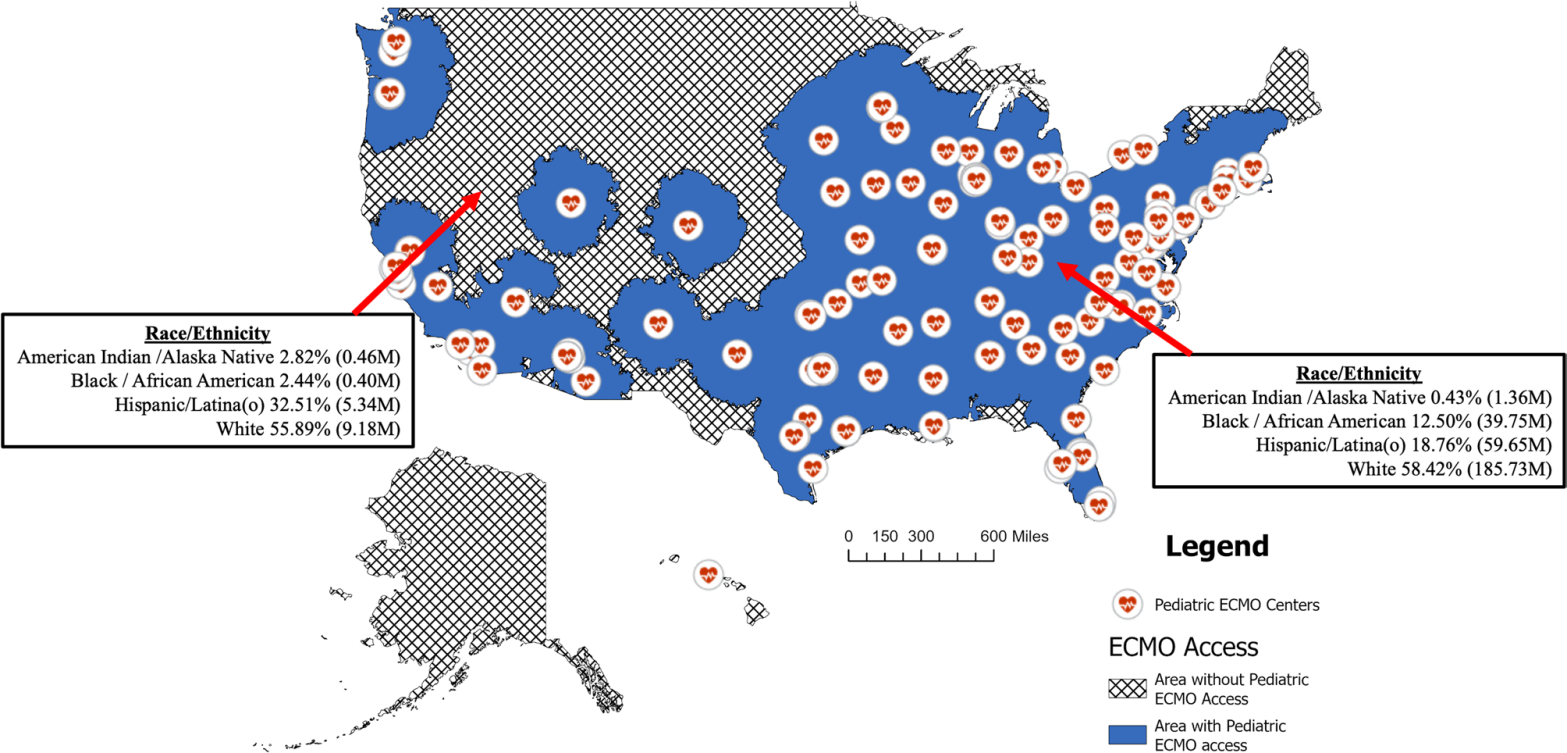
Comparison of Race/Ethnicity in regions with and without access to pediatric ECMO based on a 200-mile programmatic catchment area. Regions with and without access to Pediatric ECMO based on a 200-mile driving distance to the nearest center with Pediatric ECMO capabilities currently listed in the Extracorporeal Life Support Organization database. Abbreviations: ECMO, extracorporeal membrane oxygenation. *P* < 0.001 for all race/ethnicity proportion differences between regions

There were 150 pediatric ECMO centers identified using the ELSO Registry. Nine states did not have a pediatric ECMO program listed within the ELSO registry, and include Alaska, Idaho, Kansas, Maine, Montana, New Hampshire, North Dakota, Vermont, and Wyoming. The entirety of Montana, as well as the majority of Idaho, North Dakota, South Dakota and Wyoming were outside of the ECMO catchment area for pediatric ECMO as defined by a 200-mile driving distance. 43% of the total US land area (or 1.55 M square miles) is outside of the US catchment area for pediatric ECMO; and 4.91% of the US population (or 16,433,563 persons) does not have access to a Pediatric ECMO center. (Table 1; Fig. 1) As a whole, 25.35% of the American Indian/Alaska Native population, 1.00% of the Black / African American population, 8.22% of the Hispanic/Latina(o) population and 4.71% of the White population live outside of the total catchment area for pediatric ECMO.

**Table 1:** Comparison of the population inside and outside of the catchment area for pediatric ECMO by race/ethnicity category. Population counts and proportions for each race/ethnic category are represented, as well as for the total population

Race/Ethnicity	Population and proportion of total population inside Catchment	Population and proportion of total population outside Catchment	Proportion of Total Population Outside Catchment Area
American Indian / Alaska Native	1,363,436 (0.43%)	462,963 (2.82%)	25.35%*
Black / African American	39,748,847 (12.50%)	401,588 (2.44%)	1.00%*
Hispanic/Latina(o)	59,644,478 (18.76%)	5,342,716 (32.51%)	8.22%*
White	185,729,057 (58.42%)	9,184,602 (55.89%)	4.71*
Total	317,936,413	16,433,563	4.91%

Abbreviations: ECMO, extracorporeal membrane oxygenation. *p < 0.001 for comparison of the proportion of the race/ethnicity group within and outside of the catchment area

Of those individuals that lack access to pediatric ECMO, 462,963 (2.82%) of individuals outside of the catchment area identify as American Indian/Alaska Native compared to 1,363,436 (0.43%) in areas with access (*p* < 0.001); 404,975 individuals (2.44%) of those outside of the catchment area identify as Black/African American compared to 39,748,847 (12.50%) of those within the areas with access (*p* < 0.001); 5,342,716 (or 32.51%) of individuals outside of the catchment area identify as Hispanic/Latina(o) compared to 59,644,478 (18.76%) in the area with access (*p* < 0.001); and 9,184,602 (55.89%) of individuals outside of the catchment area identify as White compared to 185,729,057 (58.42%) that identify as White in areas with access (*p* < 0.001). (Table 1; Fig. 1) Additionally, the average distance to the nearest pediatric ECMO center for all individuals is 46.47 miles, while the distances by race/ethnicity are 161.81 miles for someone who is American Indian/Alaska Native, 26.29 miles for someone who is Black / African American, 83.29 miles for someone who is Hispanic/Latina(o) and 40.53 miles for someone who is White. (Table 2)

**Table 2:** Average distance in Miles to the nearest pediatric ECMO center by race/ethnicity category

Race/Ethnicity	Distance (US survey miles)
American Indian / Alaska Native	161.81
Black / African American	26.29
Hispanic/Latina(o)	83.29
White	40.53
Total	46.47

## Discussion

We chose to assess proximity as one actionable component of structural racism that limits access to pediatric ECMO for American Indian/Alaska Native, Black / African American, Hispanic/Latina(o) communities [17, 47, 48]. Almost half of the U.S. land area and one in 25 people lack access to pediatric ECMO. The Rocky Mountain, Great Plains and Midwest regions have the greatest dearth of pediatric ECMO programs, as does northeast New England, a portion of the Pacific Coast, Alaska and all but one Hawaii Island. Additionally, more than 1 of every 4 that identify as American Indian/Alaska Native, 1 of every 100 individuals who identify as Black / African Americans, 1 of every 12 that identify as Hispanic/Latina(o), and 1 of every 21 that identify as White lack access.

Our results indicate greater access to pediatric ECMO (95.19%) than the published geospatial analysis of access performed by Farr (2021) and adult ECMO access published by Gottula (2025) due to differences in the definitions of access used [10, 49]. We used a 200-mile catchment area, while Farr (2021) uses a 60-mile buffer and Gottula (2025) uses a 45-minute drive time [10, 49]. Our results also indicate access for a greater proportion of the population for pediatric and adult patients than that published using the European chapter of the ELSO registry by Gillon (2023), in part due to methodologic differences (they use one-, two- and three-hour drive time buffers), although differences in the distribution of ECMO centers cannot be ruled out as a contributing factor [50]. Our analysis also adds to the work by Farr (2021) by demonstrating a longer average distance to pediatric ECMO centers for American Indian / Alaska Native and Latina(o) populations as an extension of their analysis that demonstrated a similar trend using 20-, 40- and 60-mile straight-line buffers [10]. (Table 2)

While generally non-urban populations have lower access to pediatric ECMO, the designation of urban or non-urban are partly due to formal and informal policies, as well as forced relocation for racialized groups. Nonetheless even when proximity to pediatric ECMO exists, there may be other barriers to pediatric ECMO that should be explored, such as the less and delayed access to primary and specialized care for African American communities, and their increased mortality for care dependent on advanced medical technologies [33–36]. It is also worth noting that for African Americans that live in rural settings, at least some aspects of healthcare delivery may be worse than for African Americans living in urban centers [47]. 

Correcting these issues of access to lifesaving technologies is pressing, from a social justice standpoint, for populations who have broadly been excluded from access to services and those who have suffered negative health consequences from policies of exclusion and oppression. This will require investment rather than the fiscal prudency that has been used to strip hospital-based care from some racialized minority communities [37]. For American Indian and Latina(o) communities, and non-metropolitan communities as a whole, providing access to pediatric ECMO will require investment in physically extending care to areas that do not have reasonable access to it. Doing so will likely require creating guidelines and procedures for early triage of patients that might require ECMO and transport for patients that are already on ECMO support.

Transporting children on ECMO support will require further development of transport by fixed-wing airplane, a modality that has been shown effective in transporting adults and children both regionally and internationally [12, 44]. For children who are not on ECMO but might require it, improving access would likely include educating providers at remote and regional centers to assess, treat and triage pediatric patients in remote areas whose disease course might place them at a higher risk of requiring ECMO, similar to what has been advocated within the trauma literature [51]. This is important for early identification of patients that require transfer to a pediatric ECMO center, and to minimize the multiple patient transfers between remote and regional centers that can delay a patient getting to a pediatric ECMO center. This may include establishing relationships between remote centers and pediatric ECMO centers for the collaborative assessment of children, and potentially predictive modeling to discriminate between those patients that will likely require pediatric-specific critical care that could be handled at a regional center, versus those that are more likely to require ECMO [51–54]. 

Limitations to this analysis include the fact that driving distance does not account for air transport, a developing mode of transport for many ECMO programs, though one limited by the severity of illness in these patients and the physiologic implications of higher altitude. For instance, our analysis defined only one of the Hawaiian Islands as having pediatric ECMO access. Further defining of access for individual programs should include hospital affiliations and common methods of transport. Given its current limited use as noted in the methods, we do not believe that it meaningfully impacts the current analysis, but it could as air transport increases in use. Also, this analysis focuses on U.S. national racial geographies. As noted in the brief discussion of migration to the United States from Latin America, the obligation of nations that benefited from colonialism to extend resources like medical technologies to those that continue to suffer the social, political and economic consequences of colonialism is a necessary discussion not addressed through our analysis. Future studies should also look at how other elements of structural racism, like income, cost, insurance status, English proficiency and provider bias, impact access to pediatric ECMO.

## Conclusion

The introduction of ECMO as a standard of care risks further worsening access to this lifesaving therapy for already marginalized populations, particularly because of its cost and the technological expertise required to provide it. Extending this, or any standard of care medical technology, to communities that have a lower likelihood of gaining access to it should be a priority, recognizing that strategies will differ from population to population. In doing so, we must recognize that feasibility did not factor into colonization, slavery, or forced relocation, and achieving equity involves investment of capital in the provision of standard of care health services to communities that lack them regardless of the cost associated with it. Federal, state, and institutional resources should collectively be used to create the necessary infrastructure for this extension of pediatric ECMO and other high technology facets of medical care given how these oppressive structures have benefited nation, state and institution building. Future qualitative studies can survey programs to determine their individual catchments for a more granular analysis, integrate other structural inequities associated with lack of access, and should look at how access impacts mortality inside and outside of catchment areas.

## Data Availability

No datasets were generated or analysed during the current study.

## Abbreviations

ECMO: Extracorporeal membrane oxygenation
ELSO: Extracorporeal life support organization registry
